# Prospective observational cohort study of Health Related Quality of Life (HRQOL), chronic foot problems and their determinants in gout: a research protocol

**DOI:** 10.1186/1471-2474-13-219

**Published:** 2012-11-13

**Authors:** Priyanka Chandratre, Christian Mallen, Jane Richardson, Keith Rome, Joanne Bailey, Rajvinder Gill, Samantha Hider, Jane Mason, Zoe Mayson, Sara Muller, Charlotte Purcell, Jennifer Titley, Simon Wathall, Irena Zwierska, Edward Roddy

**Affiliations:** 1 Arthritis Research UK Primary Care Centre, Keele University, Staffordshire, ST5 5BG, UK; 2 Health and Rehabilitation Research Institute and School of Podiatry, AUT University, Auckland, 0627, New Zealand

**Keywords:** Gout, HRQOL, Foot, Patient experience, Prospective cohort, Primary care

## Abstract

**Background:**

Gout is the commonest inflammatory arthritis affecting around 1.4% of adults in Europe. It is predominantly managed in primary care and classically affects the joints of the foot, particularly the first metatarsophalangeal joint. Gout related factors (including disease characteristics and treatment) as well as comorbid chronic disease are associated with poor Health Related Quality of Life (HRQOL) yet to date there is limited evidence concerning gout in a community setting. Existing epidemiological studies are limited by their cross-sectional design, selection of secondary care patients with atypical disease and the use of generic tools to measure HRQOL. This 3 year primary care-based prospective observational cohort study will describe the spectrum of HRQOL in community dwelling patients with gout, associated factors, predictors of poor outcome, and prevalence and incidence of foot problems in gout patients.

**Methods:**

Adults aged ≥ 18 years diagnosed with gout or prescribed colchicine or allopurinol in the preceding 2 years will be identified through Read codes and mailed a series of self-completion postal questionnaires over a 3-year period. Consenting participants will have their general practice medical records reviewed.

**Discussion:**

This is the first prospective cohort study of HRQOL in patients with gout in primary care in the UK. The combination of survey data and medical record review will allow an in-depth understanding of factors that are associated with and lead to poor HRQOL and foot problems in gout. Identification of these factors will improve the management of this prevalent, yet under-treated, condition in primary care.

## Background

Gout is the most prevalent inflammatory arthropathy, affecting around 1.4% of the adult population in the UK [1]. It is caused by monosodium urate (MSU) crystal deposition in and around joints once the physiological saturation threshold in body tissues for uric acid is exceeded. The most commonly affected joints are the first metatarsophalangeal joint (1^st^ MTPJ), mid foot and ankle. The first acute attack affects the 1^st^ MTPJ in 56-78% of the patients with 90% having acute gout of the great toe at some point in their disease course [2] yet chronic foot problems are also common in people with gout. Hallux valgus deformity and chronic pain in the great toe are more common in people with gout than age and gender-matched controls [3]. A small hospital-based study has shown more frequent gait impairment and foot-related functional problems in patients with gout than in those without [4]. There is little evidence from a primary care perspective about the potential long-term consequences of gout for the foot.

Gout also has an adverse impact on patients’ health related quality of life (HRQOL) [5,6] and emotional, social and physical functioning, resulting in significant disability. Factors directly related to gout symptoms such as frequency and severity of acute attacks as well as those related to disease complications and adverse effects of gout treatment, all potentially contribute to impaired HRQOL. Cross-sectional epidemiological studies in primary care have shown that gout has an independent association with impaired HRQOL, particularly affecting the physical domain, after adjustments for co-morbidities such as osteoarthritis, renal and cardiovascular disease [6,7]. ‘Treatment failure’ gout within a hospital-based cohort has also been found to have a significant impact on patient HRQOL and disability, especially in the realm of physical functioning [5]. The same cohort study demonstrated that the patients’ perception of disease severity correlated more closely with HRQOL than the physicians’ assessment of disease severity. Patients and healthcare providers often have different perspectives of what constitutes optimal management of gout [8]. Whilst physicians regard pharmacological treatment of gout to be effective, most patients discontinued treatment due to adverse or no positive effects, treatment-induced flares and financial constraints [8]. A recent qualitative study [9] on the impact of gout highlighted the lack of understanding and the stigma associated with this condition which often leads to under-reporting of symptoms. This in turn can lead to suboptimal treatment despite disease severity.

These findings are not surprising given that, until recently, there has been little published work on the implications of gout in terms of morbidity and mortality as well as associated healthcare utilisation and costs [10]. The majority of gout is managed within the primary care setting, yet most of the research to date has taken place in secondary care which may deal with more complex and atypical presentations including those who have failed to respond to or not tolerated standard therapies. Therefore the applicability of such data is questionable in the wider community setting. Existing epidemiological studies have had limitations such as small sample size, cross-sectional design and the use of generic rather than disease-specific instruments such as the Gout Impact Scale (GIS) to measure HRQOL [11]. Little is known about the changes in HRQOL in gout patients due to the lack of longitudinal follow-up.

Hence there is a need for a prospective observational cohort study in primary care which incorporates patient-reported outcomes (PRO) to assess long-term outcome and consequences of gout, focusing particularly on HRQOL and foot problems. Improving understanding of which factors predict outcome would help substantiate indications for urate-lowering therapy (ULT) and identification of patients at which this should be targeted

### Objectives of the study

1. To describe the spectrum of HRQOL in patients with gout and its distribution by demographic, socio-economic and anthropometric characteristics.

2. To describe the prevalence, onset, persistence and progression of chronic foot problems in gout over 3 years.

3. To examine:

a) Cross-sectional associations between poor HRQOL and gout disease characteristics and treatment, chronic foot problems, co-morbidities, and psychosocial factors in gout.

b) Change in HRQOL in gout over 3 years and determine which of the associated factors may predict deterioration or recovery.

## Methods

### Design

A primary care-based prospective cohort study with linked medical record review. All phases of the study have been approved by the North West-Liverpool East Research Ethics Committee (Reference number 12/NW/0297).

### Sampling frame

#### Inclusion criteria

Aged >18 years.

Registered with 30 general practices in the West Midlands, UK.

Read code consultation for gout or a prescription for colchicine or allopurinol during the preceding two years.

#### Exclusion criteria

Under 18 years of age.

Vulnerable groups – e.g. significant cognitive impairment, severe enduring mental illness, active malignancy or other terminal illness.

Those who are unable to complete the questionnaires in English.

### Data collection time points

**Figure 1:**
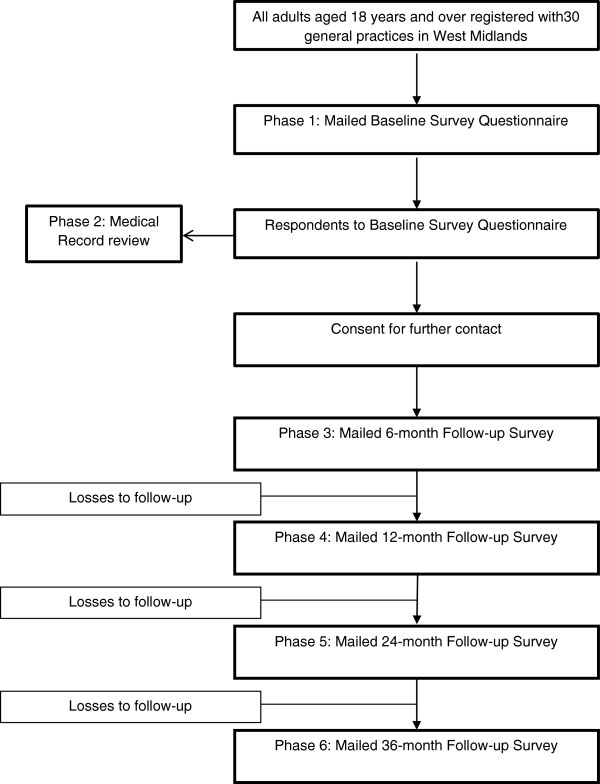
Flowchart of study procedure.

The different phases of the study are illustrated in Figure 1.

#### Phase 1: baseline postal questionnaire survey

##### Patient identification

Staff from the West Midlands North Primary Care Research (WMN PCR) will conduct a single electronic search of the primary care records in participating practices to identify patients with Read codes for a consultation for gout or a prescription for colchicine or allopurinol within the last two years. The Read codes used by the Arthritis Research UK Primary Care Centre (ARUKPCC) to define gout are listed in Table 1. The WMN PCR team members will screen the mailing lists (prior to mailing) for patient deaths and departures from the practice to ensure that patients are not inappropriately contacted. The lead general practitioner (GP) at each practice will be invited to identify potentially vulnerable patients to be excluded.

**Table 1:** Read Codes used to identify consultations with gout in primary care

**Code**	**Term**
C34	Gout
N023	Gouty arthritis
EGTON 227	Gout NOS
OX2740G	Gout Acute/ox
1443	H/O: gout
EMISR4QG01	Gouty tophi + Gout NOS
2D52	O/E - auricle of ear - tophi
669	Gout monitoring

##### Initiating patient contact

All eligible patients will be sent a study pack from their GP containing a letter of invitation, participant information sheet (PIS), a pre-paid return envelope and a baseline self-completion questionnaire which will also include a consent form asking for consent for further contact and review of their medical records. Potential participants will be provided with a contact name and telephone number should they have any queries about the study. Patients will be informed that they are under no obligation to participate and that if they decline their normal clinical care will not be affected in any way. Participants will be asked to return completed questionnaires, and upon receipt by the research centre, the response will be recorded against a unique patient number in a mailing database.

##### Non-responders to mailed baseline study pack

After two weeks, those who have not responded will be sent a reminder postcard from their GP. After a further two weeks, a reminder letter with repeat baseline questionnaire will be sent to those who have yet to respond (4 weeks after the first questionnaire). Those who fail to respond after all three baseline mailings will be assumed not to have consented to the study and will not be contacted again.

##### The questionnaire

The questionnaire will be divided into 7 main sections

a) Gout symptoms and treatment.

b) The impact of gout on daily life.

c) General health (including co-morbidities and measures of physical function).

d) Measures of anxiety and depression.

e) Foot and other joint problems.

f) Occupational characteristics.

g) Socio-economic and demographic characteristics.

Details of the conceptual domains, operational definitions and empirical measures are provided in Table 2. The completed baseline questionnaires will have the responses securely stored in the study database.

**Table 2:** Questionnaire items

**Conceptual domain**	**Operational definition**	**Empirical measure**	**Number of items**	**Time point**
**Section A: About Gout**				
Gout frequency	No. of attacks in the last 12 months/since last contact	Numerical rating scale 0- ≥ 5	1	All
Age at diagnosis	Age in years	Numerical free text box	1	BL
Acute attack of gout	Acute episode at time of questionnaire	Yes/No	1	All
Allopurinol	Reported use of allopurinol	Yes/No	1	All
	Current daily dose of allopurinol	Nine daily dose options: 50 mg-900 mg	1	All
**Section B: How gout affects your life**				
Gout concern, wellbeing, productivity, convenience and satisfaction	Gout Impact Scale [11]	5-item Likert scale	18	All
Illness perception	Modified Illness perception questionnaire [12]	5-item Likert scale	4	BL, 12 months, 36 months
**Section C: General Health**				
Physical function	SF36 Physical function sub-scale (PF10) [13]	3-item Likert scale	10	All
	Health Assessment Questionnaire Disability Index [14]	4-item Likert scale	17	All
Co-morbidities	Diabetes mellitus, Renal failure, renal calculi, Cerebrovascular accident, Transient ischaemic attacks, ischaemic heart disease, hyperlipidaemia	Yes/No	9	BL
**Section D: How you feel**				
Depression	Patient health questionnaire (PHQ 9) [15]	4 point Likert scale	16	BL, 12 months, 36 months
Anxiety	Generalised anxiety disorder questionnaire (GAD) [16]			
**Section E: Foot and other joint problems**				
Hallux valgus	Self-completed line drawings [17]	5 line-drawings for each foot depicting increasing severity of hallux valgus	2	BL, 12 months, 36 months
Pain	Pain in the hands, hips, knees and feet in the last year	Yes/No	4	BL, 12 months, 36 months
	Location of body pain in last 4 weeks	Self-completed body manikin [18,19]	1	BL, 12 months, 36 months
Foot pain	Foot pain, aching, stiffness in last month [20]	Frequency on 5-point Likert scale	1	BL, 12 months, 36 months
Foot pain location	Location of foot pain in last four weeks	Self-completed foot manikin [21]	1	BL, 12 months, 36 months
Foot function	Manchester Foot Pain and Disability Index [22]	Frequency on 3-point likert scale	17	BL, 12 months, 36 months
Consultation for foot problems	Consultation with GP, physiotherapy, podiatry, in last 12 months/since last contact	Yes/No	4	BL, 12 months, 36 months
**Section F: Work**				
Occupational characteristics	Current employment status	11-response options	1	BL, 12 months, 36 months
	Work absence during last 6 months due to joint/back problems	Yes/No	1	BL, 12 months, 36 months
	Ability to do usual job	5-response options	1	BL, 12 months, 36 months
**Section G: Demographic/socioeconomic characteristics**				
Date of birth	Date of birth	Date of birth	1	BL
Gender	Gender	Male/Female	1	BL
Anthropometric characteristics	Height	Meters or feet/inches	1	All
	Weight	Kilogram or stones/pounds	1	All
Marital status	Marital status	6-response options	1	BL
Living alone	Living alone	Yes/No	1	BL
Adequacy of income	Adequacy of income	4-response options	1	BL
Education	Higher education	Yes/No	1	BL
Ethnicity	Ethnicity	6-response options	1	BL
Life-style-characteristics	Frequency of alcohol consumption	6-response options	1	BL
	Weekly amount of beer/spirits/wine consumed	Free-text	1	BL
	Smoking status	3-response options	1	BL

##### Data entry, coding, cleaning and storage

A specific study database will be created to record responses to the questions. Data entry will be performed by dedicated trained members of the administrative team as the completed questionnaires are returned. Although they are experienced in data entry, specific training will be provided for this study. The principal investigator (PI) and study statistician will determine coding prior to data entry into the database which will provide coding options. One in ten random questionnaires will be checked by a member of the study team for the purposes of quality assurance. This information is kept by the research support co-ordinator. Only relevant members of the research team will have access to the database which is password protected. Requests for access to the data stored in this database must be made in writing, along with an analysis plan, to the Chief Investigator (CI). Questionnaires and consent sheets are securely stored in separate locations to protect patient confidentiality.

#### Phase 2: Review of general practice medical records

All participants in Phase 1 who give permission for their GP records to be accessed will have their computerised medical records tagged by a member of the WMN PCR team. The practices participating in this study are fully computerised and undergo annual audits completed by the WMN PCR team to assess the quality and completeness of the data at the practices [23]. All consultations for the 2 years prior to study entry and then prospectively for the three-year study period will be identified. The data obtained will include co-morbidities, repeat consultations for gout, prescription patterns and referral to secondary care. All patient identifiable data (name, contact details) will be removed from the medical records and the consultation data will be linked to the survey data by unique survey identifier.

#### Phase 3, 4, 5 and 6: Follow-up at 6, 12, 24 and 36 months

Follow-up surveys will be mailed at 6, 12, 24 and 36 months to all participants in phase 1 who consented to further contact. The focus of follow-up will be clinical (pain/disability severity) change and the possible determinants of this. The questionnaire will include repeated measures of general health (including generic measures of physical function), psychosocial factors, co-morbidity and gout symptoms. Non-responders to the questionnaire will be sent a reminder postcard after two weeks. Those who do not respond to the reminder postcard will be sent a repeat questionnaire, PIS and a further covering letter four weeks after the initial mailing. The WMN PCR team members will screen the mailing lists (prior to mailing) for patient deaths and departures from the practice to ensure that patients are not inappropriately contacted.

##### Sample size

Disease specific HRQOL scores will be recorded using the Gout Impact Scale at baseline, 6, 12, 24 and 36 months. In order to use the information recorded at all five points, a sample size of 882 would allow a smallest meaningful difference in HRQOL of 0.2 standard deviation units to be detected between two groups (441 subjects per group) defined in terms of frequency of gout attacks (<2 attacks, ≥2 attacks per year) using a linear mixed model (significance 0.05, power 90%, autocorrelation 0.8) [24]. Allowing for 70% response at baseline and 30% drop out over the follow-up period would require 1800 people with gout to be contacted at baseline.

### Statistical analysis

#### Baseline

Descriptive statistics will be used to assess response bias, along with the characteristics of the baseline population.

Factors associated with levels of HRQOL at baseline will be assessed using students’ t-tests chi-squared tests, and logistic regression, as appropriate.

##### Follow-up

Descriptive statistics will be used to assess attrition bias and to describe the onset, and persistence of foot problems and their characteristics.

Regression models will be used to assess the factors predicting poor HRQOL and chronic foot problems prospectively over three years.

Imputation techniques will be used to account for missing data and loss to follow up, as appropriate.

## Discussion

HRQOL is an important yet under-researched outcome measure in chronic gout. To our knowledge this is the first prospective observational cohort of gout patients in primary care in the UK which uses generic as well as gout-specific questionnaires to assess HRQOL. Through follow-up surveys and medical record review, the study investigates the occurrence and frequency of poor HRQOL, factors associated with it at baseline and predictors of poor outcome at follow-up. A limitation of the study is the identification of patients based on a clinical diagnosis of gout (the gold standard of urate crystal identification in synovial aspirate [25] is not mandatory for inclusion into the study). However, a clinical diagnosis based on the rapid onset of pain, erythema and swelling affecting the 1^st^ MTPJ in the context of hyperuricaemia is supported by the European League Against Rheumatism (EULAR) recommendations for the diagnosis of gout [25]. Potential participants will be identified either by a gout-coded primary care consultation or a prescription for allopurinol or colchicine in the study period. Other urate lowering therapies such as febuxostat and uricosuric drugs will not be included in this search strategy as both are infrequently used in UK primary care. Patients taking either drug will be identified by regular consultations. This study ultimately aims to improve the management of gout in primary care through identifying and considering factors associated with and predictive of poor outcome in a patient-centred treatment plan.

## Competing interests

The authors declare that they have no competing interests.

## Authors’ contributions

ER and CM conceived the study. All authors participated in the study design. PC, CM and ER drafted the manuscript which was approved by all authors.

## Pre-publication history

The pre-publication history for this paper can be accessed here:

http://www.biomedcentral.com/1471-2474/13/219/prepub

## References

[B1] Annemans L Spaepen E Gaskin M Bonnemaire M Malier V Gilbert T Gout in the UK and Germany: Prevalence, co-morbidities and management in general practice 2000–2005 Ann Rheum Dis 2008 67 960 966 17981913 10.1136/ard.2007.076232 PMC2564789

[B2] Roddy E Revisiting the pathogenesis of podagra: Why does gout target the foot? J Foot Ankle Res 2011 4 1 13 10.1186/1757-1146-4-13 21569453 PMC3117776

[B3] Roddy E Zhang W Doherty M Gout and nodal osteoarthritis: A case–control study Rheumatology 2008 47 5 732 733 10.1093/rheumatology/ken087 18356175

[B4] Rome K Survepalli D Sanders A Lobo M McQueen FM McNair P Functional and biomechanical characteristics of foot disease in chronic gout: A case–control study Clin Biomech 2011 26 1 90 94 10.1016/j.clinbiomech.2010.09.006 20950904

[B5] Becker MA Schumacher HR Benjamin KL Gorevic P Greenwald M Fessel J Quality of life and disability in patients with treatment-failure gout J Rheumatol 2009 36 5 1041 1048 10.3899/jrheum.071229 19332629

[B6] Roddy E Zhang W Doherty M Is gout associated with reduced quality of life? A case–control study Rheumatology 2007 46 9 1441 1444 10.1093/rheumatology/kem150 17586863

[B7] Singh JA Strand V Gout is associated with more co-morbidities, poorer health-related quality of life and higher healthcare utilisation in US veterans Ann Rheum Dis 2008 67 9 1310 1316 18178692 10.1136/ard.2007.081604

[B8] Harrold LR Mazor KM Velten S Ockene IS Yood RA Patients and providers view gout differently: A qualitative study Chronic Illness 2010 6 4 263 271 10.1177/1742395310378761 20675361 PMC3134238

[B9] Lindsay K Gow P Vanderpyl J Logo P Dalbeth N The experience and impact of living with gout: A study of men with chronic gout using a qualitative grounded theory approach J Clin Rheumatol 2011 17 1 1 6 21169857 10.1097/RHU.0b013e318204a8f9

[B10] Wu EQ Patel PA Yu AP Disease-related and all-cause health care costs of elderly patients with gout J Manag Care Pharm 2008 14 164 175 18331118 10.18553/jmcp.2008.14.2.164 PMC10437756

[B11] Hirsch JD Lee SJ Terkeltaub R Khanna D Singh J Sarkin A Evaluation of an instrument assessing influence of gout on health-related quality of life J Rheumatol 2008 35 12 2406 2414 10.3899/jrheum.080506 18925685

[B12] Moss-Morris R Weinman J Petrie K Horne R Cameron L Buick D The revised illness perception questionnaire (IPQ-R) Psychol Health 2002 17 1 1 16 10.1080/08870440290001494

[B13] Ware JE Jr Sherbourne CD The MOS 36-item short-form health survey (SF-36). Conceptual framework and item selection Med Care 1992 30 473 483 10.1097/00005650-199206000-00002 1593914

[B14] Bruce B Fries JF The Stanford health assessment questionnaire (HAQ): A review of its history, issues, progress, and documentation J Rheumatol 2003 30 1 167 12508408

[B15] Kroenke K Spitzer RL Williams JBW The PHQ-9 J Gen Intern Med 2001 16 9 606 613 10.1046/j.1525-1497.2001.016009606.x 11556941 PMC1495268

[B16] Spitzer RL Kroenke K Williams JBW Lowe B A brief measure for assessing generalized anxiety disorder: The GAD-7 Arch Intern Med 2006 166 10 1092 1097 10.1001/archinte.166.10.1092 16717171

[B17] Roddy E Zhang W Doherty M Validation of a self-report instrument for assessment of hallux valgus Osteoarthr Cartilage 2007 15 9 1008 1012 10.1016/j.joca.2007.02.016 17387024

[B18] Hunt I Silman A Benjamin S McBeth J Macfarlane G The prevalence and associated features of chronic widespread pain in the community using the ‘Manchester’ definition of chronic widespread pain Rheumatology 1999 38 275 279 10.1093/rheumatology/38.3.275 10325667

[B19] Lacey R Lewis M Jordan K Jinks C Sim J Inter-rater reliability of scoring pain drawings in a self-report health survey Spine 2005 30 455 458 10.1097/01.brs.0000153393.82368.6b 16103839

[B20] Dufour AB Broe KE Nguyen US Gagnon DR Hillstrom HJ Walker AH Kivell E Hannan MT Foot pain: is current or past shoe wear a factor? Arthritis Rheum 2009 61 1352 1358 10.1002/art.24733 19790125 PMC2761974

[B21] Garrow AP Silman AJ Macfarlane GJ The Cheshire foot pain and disability survey: A population survey assessing prevalence and associations Pain 2004 110 1–2 378 384 15275789 10.1016/j.pain.2004.04.019

[B22] Garrow AP Papageorgiou AC Silman AJ Thomas E Jayson MI Macfarlane GJ Development and validation of a questionnaire to assess disabling foot pain Pain 2000 85 107 113 10.1016/S0304-3959(99)00263-8 10692609

[B23] Porcheret M Hughes R Evans D Jordan K Whitehurst T Ogden H Data quality of general practice electronic health records: The impact of a program of assessments, feedback, and training J Am Med Inform Assoc 2004 11 1 78 86 14527973 10.1197/jamia.M1362 PMC305461

[B24] Diggle P Analysis of longitudinal data 2002 2 Oxford: Oxford University Press

[B25] Zhang W Doherty M Pascual E EULAR evidence based recommendations for gout - Part I diagnosis: report of a task force of the standing committee for international clinical studies including therapeutics (ESCISIT) Ann Rheum Dis 2006 65 1301 1311 10.1136/ard.2006.055251 16707533 PMC1798330

